# Solvent Composition Drives the Rebinding Kinetics of Nitric Oxide to Microperoxidase

**DOI:** 10.1038/s41598-018-22944-z

**Published:** 2018-03-27

**Authors:** Padmabati Mondal, Markus Meuwly

**Affiliations:** Department of Chemistry, University of Basel, Klingelbergstrasse 80, 4056 Basel, Switzerland

## Abstract

The rebinding kinetics of NO after photodissociation from microperoxidase (Mp-9) is studied in different solvent environments. In mixed glycerol/water (G/W) mixtures the dissociating ligand rebinds with a yield close to 1 due to the cavities formed by the solvent whereas in pure water the ligand can diffuse into the solvent after photodissociation. In the G/W mixture, only geminate rebinding on the sub-picosecond and 5 ps time scales was found and the rebinding fraction is unity which compares well with available experiments. Contrary to that, simulations in pure water find two time scales – ~10 ps and ~200 ps - indicating that both, geminate rebinding and rebinding after diffusion of NO in the surrounding water contribute. The rebinding fraction is around 0.63 within 1 ns which is in stark contrast with experiment. Including ions (Na and Cl) at 0.15 M concentration in water leads to rebinding kinetics tending to that in the glycerol/water mixture and yields agreement with experiments. The effect of temperature is also probed and found to be non-negligible. The present simulations suggest that NO rebinding in Mp is primarily driven by thermal fluctuations which is consistent with recent resonance Raman spectroscopy experiments and simulations on MbNO.

## Introduction

Heme-containing proteins belong to a versatile class of macromolecules present in all types of living organisms and are of central importance in different biological processes. The reactivity and selectivity of heme proteins depends largely on the ability of the heme-iron to switch from one oxidation state to the other usually upon binding of a diatomic ligand including carbon monoxide (CO), nitric oxide (NO), or oxygen (O_2_). This is relevant during process such as respiration, catalysis or signalling. Therefore, it is important to understand at a molecular level how ligand binding, unbinding and migration occur in heme-containing proteins.

Microperoxidases (Mps) are small, highly soluble proteins, produced due to the proteolytic digestion of cytochrome c (Cytc) and are generally 8–11 amino acids long^1^. Nitric oxide binds to all heme-containing proteins and is an important intra and inter cellular messenger with roles in the cardiovascular, immune and nervous system of mammals^2,3^. The ligand is synthesized by the oxidation of L-ariginine catalyzed by the heme-proteins NO-synthases with intermediate formation of N-hydroxy-L-arginine (NOHA). NOHA is known to produce NO in the presence of Mps and hydrogen peroxidase where Mp acts as a catalyst and forms the Mp-Fe-NO complex^4^. Therefore, understanding the rebinding kinetics of NO to Mp is also relevant for the catalytic efficiency of Mps in NO synthesis. Mps are also known to catalyze several reactions like Cytochrome P450 type oxygen transfer, para-hydroxylation of aniline, S-oxidation of thioether, N- and O-dealkylation of aromatic amines or ether, Nitric oxide synthesis and also acts as NO scavenger^4–6^.

The rebinding kinetics of heme proteins has been studied extensively experimentally and theoretically^7–13^. This includes rebinding kinetics of NO, CO, O_2_ to myoglobin (Mb), Haemoglobin (Hb) or Cytc. Recently, the rebinding kinetics of NO to Mp-8 was experimentally studied by time-resolved vibrational spectroscopy where very fast (pico-second) geminate rebinding of NO with 100% rebinding efficiency was observed^14,15^. Unlike for MbNO and MpCO (which is non-exponential), they found the rebinding kinetics for MpNO to be single-exponential which dismissed the diffusion model for the ligand^16^ or the distribution of the rebinding barriers^17,18^ (suggesting non-exponential rebinding kinetics) and concluded that rebinding is driven by a mechanism by which the unpaired electron of NO “harpoons” the iron back into the heme-plane^14,19^. On the other hand, recent experiments using time-resolved resonance Raman and femtosecond transient absorption spectroscopy measurements^20^ and simulations^21^ on MbNO suggest that the kinetics can be explained by thermal effects only whereby the potential energy surfaces for the ^2^*A* and ^4^*A* states lead to an insignificant rebinding barrier, in agreement with experimental observations.

**Figure 1:**
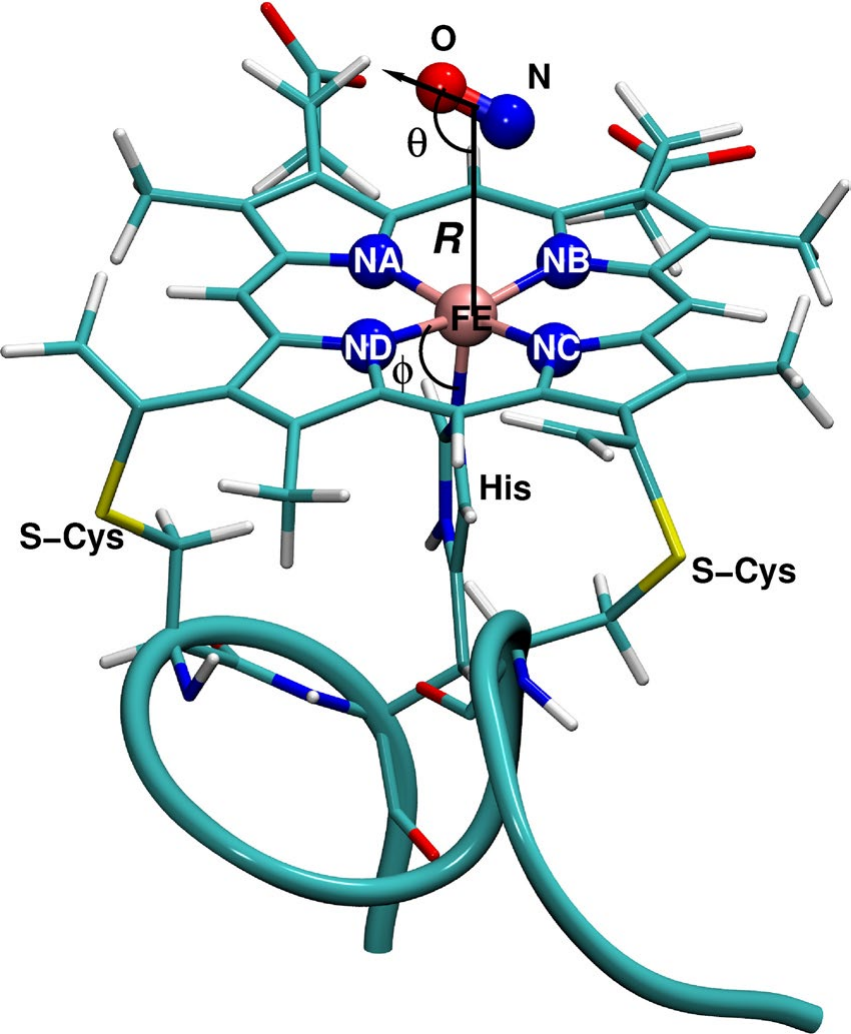
The structure of Mp-9 with deligated NO. The heme, His and Cys groups are shown in licorice representation. Fe and four nitrogens of the heme group are shown in VDW representations and NO is shown in CPK representation. The internal coordinates (*R*, *θ*, *ϕ*), specifying the Fe-COM_NO_ distance, Fe-COM_NO_-O angle and iron doming angular coordinate, are also shown.

Being smaller in size than Mb, Hb or Cytc, Mps are a prototypical example for ligand binding to a coordinated heme-unit which can be extensively studied using advanced atomistic simulation methods such as reactive molecular dynamics. The influence of the solvent in biomolecular reactions in solution has been studied previously and was found to affect both, their thermodynamics and kinetics^22–25^. As the active site is solvent-exposed in Mp (see Fig. 1) as opposed to Mb, Hb or Cytc, ligand (re)binding is directly influenced by the presence, structure and dynamics of the surrounding solvent and its composition. While several previous studies focussed on the effect of shape, volume and polarity of the distal heme pocket to the migration and stability of the ligand-binding of Mb, Hb, Cytc^26–28^, the effect of solvent (especially in view of solvent compositions) on the ligand-rebinding of the solvent-exposed heme protein Mp at the atomistic level remains unexplored. Such effects are of even greater relevance in the context of molecular crowding which occurs in realistic biological environments^29–32^. The presence of ions in the solvent can modulate the properties of the environment akin to crowding and may directly influence reactive processes like protein-ligand (re)binding. *In vivo* experiments especially in cells can sometimes face technical challenges due to molecular crowding. Therefore, molecular dynamics simulations studies are a meaningful way to obtain atomistic level information into the effect of molecular crowding on biological processes and environments.

In this work, we studied the rebinding kinetics of NO to Mp using reactive force fields (FFs) in different environments e.g. glycerol/water (G/W) mixture, pure water and water plus ions. As the solvent environment may play an important role in controlling reactions at the solvent-exposed site of Mps, methods which allow to study chemical reactivity (binding/unbinding) and running multiple trajectories over extended time scales are of particular interest. The reactive force fields for the photo-excitation from the bound (^2^A) and unbound (^4^A) states and rebinding (from the ^4^A to the ^2^A state) were obtained by 3-dimensional *ab initio* potential energy surface (PES) with the reproducing kernel Hilbert space (RKHS) potential^33,34^. Based on these PESs and the multisurface adiabatic reactive molecular dynamics (MS-ARMD) method^35^ to connect them, the rebinding kinetics of MpNO was determined in different solvent environments. The effect of temperature on the rebinding kinetics was also investigated.

## Results and Discussion

### The Intermolecular Potential

**Figure 2:**
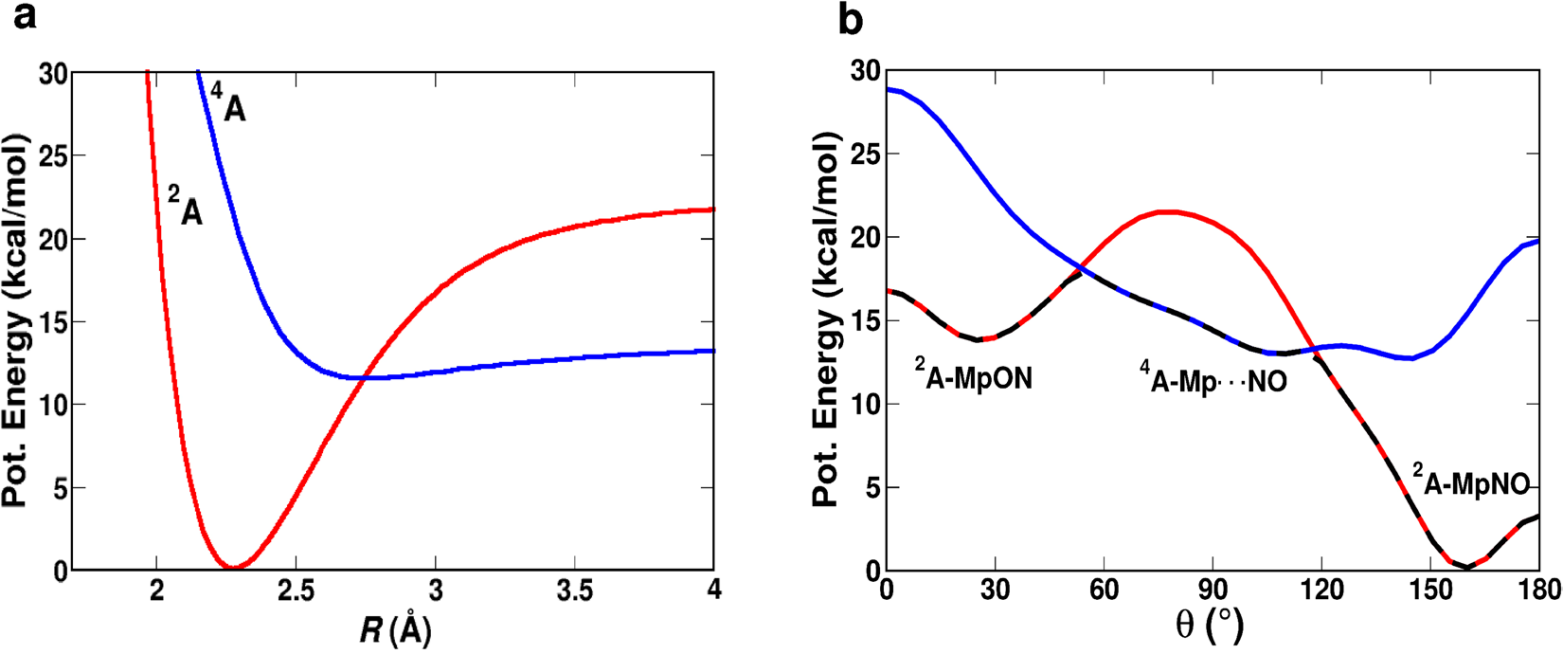
Potential energy cuts (**a**) along *R* (for *θ* = 160°) and (**b**) along *θ* (for *R* = 2.4 Å) using the RKHS potential in the gas phase for a shift value of Δ = 5 kcal/mol. The red and blue traces correspond to the ^2^*A* and ^4^*A* states, respectively. The black dashed line in panel b represents the lowest adiabatic cut along *θ*.

First, the intermolecular interactions for the adiabatic PESs for different situations are considered. The bare RKHS PESs used in this work, are shown in Fig. 2 for the radial coordinate *R* and along the angular degree of freedom *θ* (see Fig. 1). The radial cut for *θ* = 160° exhibits a deep minimum at *R* = 2.37 Å with a binding energy of 21 kcal/mol. This state corresponds to the thermodynamically stable FeNO state. The ^2^*A* surface exhibits a second minimum for *θ* = 27° and *R* = 2.48 Å which corresponds to the FeON conformer with a binding energy of 6 kcal/mol. For the excited ^4^*A* state a predominantly repulsive potential is found (blue line in Fig. 2a). Nevertheless, a faint minimum is also present on the ^4^*A* state. The angular cut (Fig. 2b) along *θ* (for *R* = 2.4 Å) shows that the two bound state minima (^2^*A*-MpON and ^2^*A*-MpNO) are separated by 14 kcal/mol. It is seen in Fig. 2b that starting from ^2^*A* MpON, the system switches to the ^4^*A* Mp$\cdots$NO (at *θ* = 55°) and then again returns to the ^2^*A* MpNO state around 120°. Therefore, the transition from MpON state to MpNO (isomerisation), needs to overcome two barriers; first, from the ^2^*A* MpON to the ^4^*A* Mp$\cdots$NO (3.5 kcal/mol) and second, from the ^4^*A* Mp$\cdots$NO to the ^2^*A* MpNO (1 kcal/mol).

**Figure 3:**
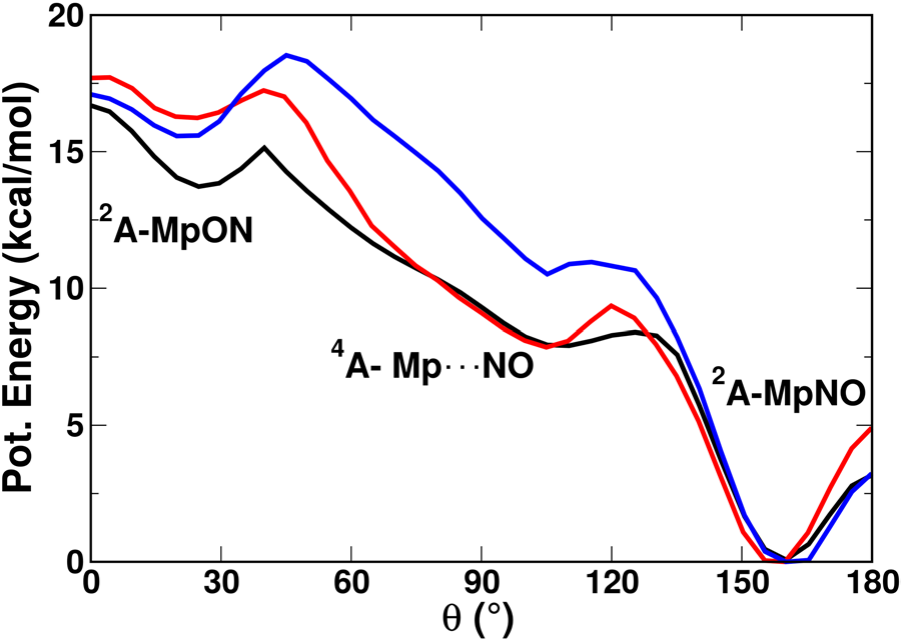
Comparison of the lowest adiabatic potential energy cuts along the angle *θ* (for *R* = 2.4 Å) calculated using the fitted RKHS plus FF potential for His-heme-NO system in gas phase (black), pure water (red) and in 90% G/W mixture (blue) for Δ = 7.5 kcal/mol.

Next, the adiabatic surfaces for the Histidine-heme-NO system in the gas phase, in pure water and in the mixed G/W mixture along the angular coordinates were determined. For the system in the gas phase the adiabatic surface is reported as the black line in Fig. 3 (for Δ = 7.5 kcal/mol). Because only the energetics along the (*R*, *θ*) coordinates of the NO molecule are of interest, only a minimal solvent environment was considered. In pure water, 11 water molecules were included whereas for mixed G/W, 4 glycerol and 3 water molecules were used.

The scans along *θ* describe the isomerization pathway between FeNO and FeON. As Fig. 3 illustrates the shape of the PES, the barriers between neighbouring minima and their relative stabilization on the lowest adiabatic potential energy cut along the angle *θ* depend on the type of the environment. Broadly speaking, starting from the the MpON (^2^*A*) structure (*θ* = 30°), the system switches to the Mp$\cdots$NO (^4^*A*) state (around *θ* = 55°) and then back to the ^2^*A* state to end up in the MpNO configuration with *θ* = 160°. While in all environments the MpON metastable state is present, the barriers towards the MpNO state differ. In the gas phase, the barriers are 1.2 kcal/mol and 1 kcal/mol at *θ* = 45° and *θ* = 130°, respectively (black trace in Fig. 3) whereas in pure water and in the G/W mixture the barrier increases to 2 kcal/mol and 3 kcal/mol at *θ* = 45° and 2 kcal/mol and 1.5 kcal/mol at *θ* = 130°, respectively.

### Photodissociation of MpNO in G/W mixture

**Figure 4:**
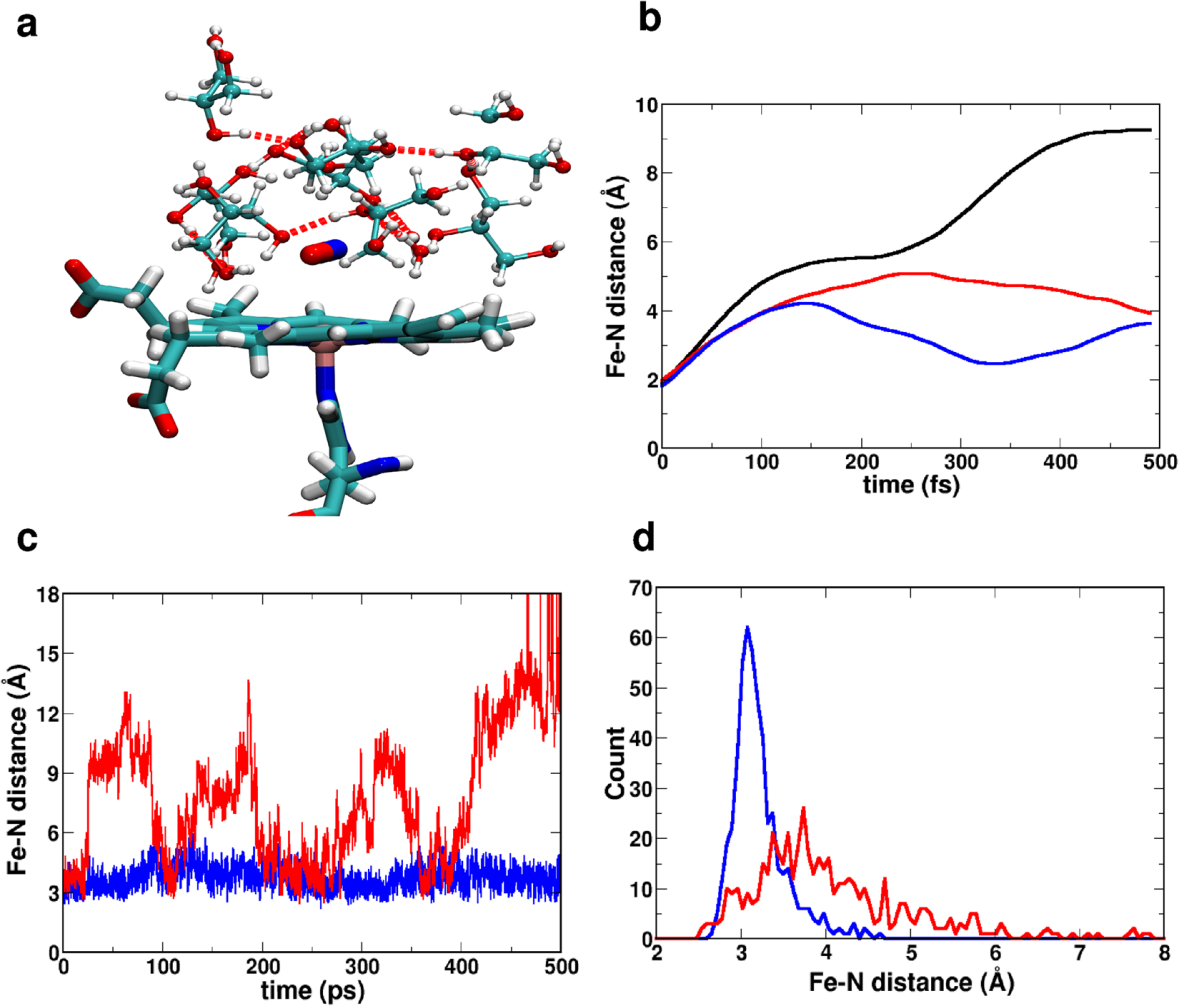
(**a**) Caged, H-bonded structure around the photodissociated NO ligand (licorice) formed in the G/W mixture. (**b**) The Fe-N distance for a typical photodissociation trajectory of 500 fs in G/W mixture (blue) compared to the dynamics in pure water (red) and in the gas phase (black). (**c**) Dynamics on the ^4^A state. Comparison of Fe-N distances for deligated NO in water (red) and in the G/W mixture (blue). The caged dynamics in the G/W mixture differs appreciably from the escape-type dynamics in pure water. (**d**) Final distribution of Fe-N distances for 500 independent trajectories after 500 fs of dynamics following photo dissociation. Blue for G/W and red for the dynamics in pure water.

Next, the dynamics of photodissociated NO from MpNO was studied in a 90/10 G/W mixture (see Fig. 4a). Because G/W forms caged structures due to strong G/G and G/W intermolecular hydrogen bonding^36^, liberating NO from MpNO is likely to lead to constrained dynamics of the ligand, akin to the situation in myoglobin or hemoglobin^19,20,37,38^. This is indeed the case as Fig. 4b,c (blue line) demonstrate.

When NO is photodissociated (500 fs) from MpNO in the gas phase, the ligand follows a trajectory in which the Fe-N separation increases monotonically, see black trace Fig. 4b. This changes with water (red) or G/W (blue) surrounding the ligand upon photo-dissociation in that the translational motion is clearly hindered and damped compared to the situation in the gas phase. Following the ligand trajectory for 500 ps on the excited ^4^*A* state demonstrates the difference between the dynamics in water and in the G/W mixture. In G/W the photodissociated ligand is trapped near the heme-iron atom whereas in pure water the ligand makes several attempts to diffuse into the solvent (Fe-N distance > 8 Å). After each unsuccessful escape NO returns to a position close to the heme-iron before it finally escapes after 400 ps. In these simulations the system is not allowed to rebind to the ^2^*A* state. Rather, the dynamics takes place entirely on the ^4^*A* PES. Finally, for an ensemble of 500 trajectories the probability distribution of the Fe-N separation after 500 fs of photo-dissociation dynamics is shown in Fig. 4d. In a G/W mixture (blue) the ligand remains close to the active site whereas in pure water (red) the distribution is much broader and peaks at 3.9 Å compared to 3.0 Å for simulations in G/W.

### Rebinding Kinetics of MpNO in G/W mixture

Next, the rebinding kinetics following photo-dissociation was studied. For this, the simulations were run on the mixed, reactive PES allowing rebinding (and unbinding) including both states (^2^A and ^4^A) as described in the Methods Section. A total of 500 rebinding molecular dynamics trajectories were run starting from the initial conditions generated as described in the Methods. In the following, the trajectories which rebind are referred to as “reactive” whereas those which do not rebind within 1 ns are considered as “non-reactive”.

**Figure 5:**
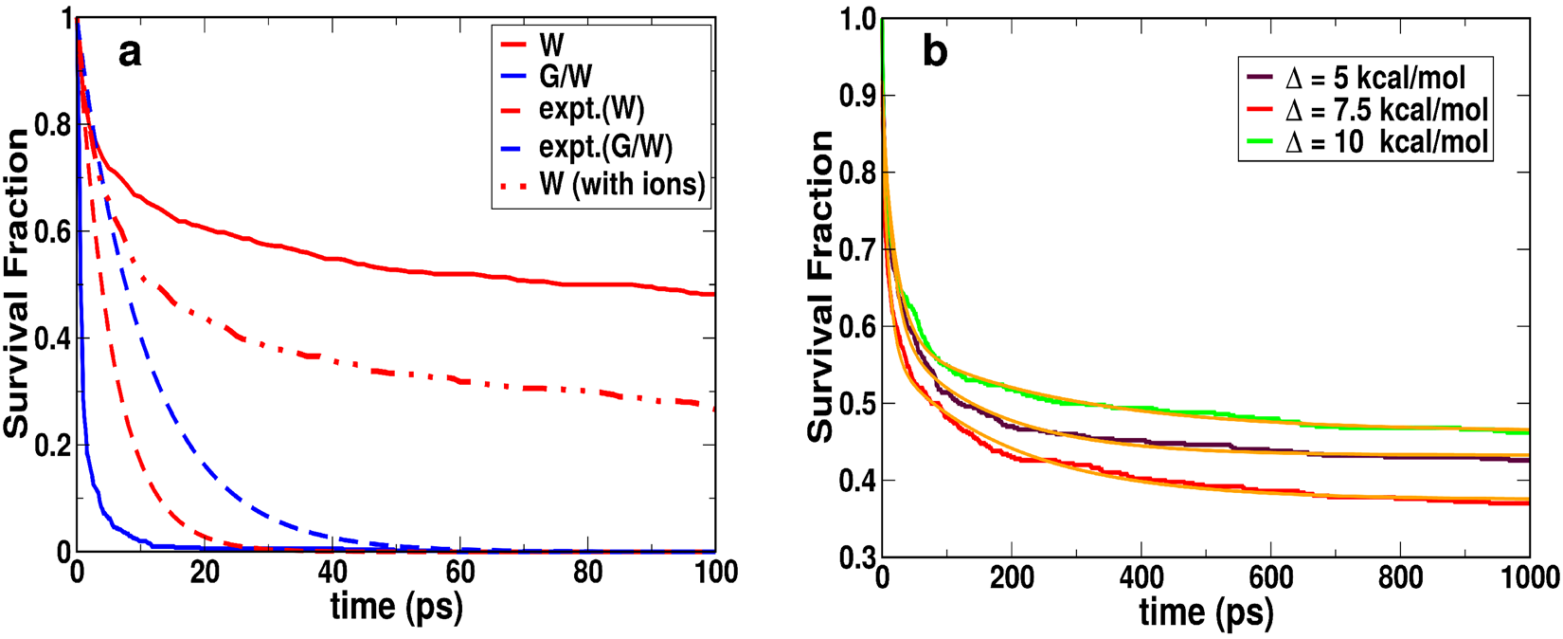
(**a**) Comparison of rebinding kinetics of NO to Mp-9 in water (red) and in G/W mixture (blue) for Δ = 7.5 kcal/mol. The dashed lines are the experimental rebinding curves for NO rebinding to Mp-8 in D_2_O^37^ and in G/W mixture^14^, respectively. The red dotted-dashed line is the simulated rebinding curve for MpNO in water with 0.15 M NaCl ions. (**b**) Survival fraction for 1 ns for the rebinding of MpNO in pure water for Δ = 5 (maroon), 7.5 (red), 10 (green) kcal/mol. The solid orange lines correspond to bi-exponential fitting with static components.

Figure 5a and Table 1 summarize the experimental and computed data for the rebinding kinetics of MpNO in G/W (compared to MpNO in pure water). The rebinding fraction in the G/W mixture was found to be close to unity, irrespective of the value of Δ (see Methods). The rebinding kinetics can be described with a bi-exponential decay including a sub-picosecond phase and a time scale of around 5 ps both of which correspond to the geminate rebinding. The amplitude of the fast component is around 80% compared to 20% for the slow component. The rebinding fraction of 100% agrees well with experiment whereas the rebinding time scale in the experiment is 8 ±1 ps^15^ and 11 ps^14^ which is about a factor of 1.5–2 longer compared with the computations. Probably the sub-ps timescale found in the simulations is difficult to be resolved in experiments. It is instructive to compare these results with the situation in MbNO where invariably two rebinding time scales are found: one on the 5 to 25 ps time scale and another one on the 100 to 300 ps time scale depending on the actual experiment^12,21,37,39–42^. This compares with computed rebinding times around 10 and 100 ps^21^. Hence, a difference of a factor of two between experimentally observed and computed rebinding time scales is realistic and expected.

**Table 1:** Parameters for the tri-exponential fit of the survival fraction from rebinding trajectories for the MpNO in G/W mixture, in pure water and in water + ions using RKHS potential with different shift values.

Solvent	Δ (kcal/mol)	*T* (K)	*t*_sim_ (ns)	Rebinding fraction	*a*_1_	*τ*_1_ (ps)	*a*_2_	*τ*_2_ (ps)	*a*_3_
G/W	7.5	300	0.1	0.99	0.89	0.55	0.11	4.1	
	10.0	300	0.1	1.0	0.81	0.64	0.19	4.6	
exp^14^		300		1.0				11.0	
pure water	5.0	300	1.0	0.58	0.30	21.7	0.12	267.1	0.47
	5.0	300	0.1	0.48	0.24	6.1	0.29	74.1	0.44
	7.5	300	1.0	0.63	0.35	10.7	0.20	192.3	0.37
	7.5	300	0.1	0.52	0.24	3.1	0.25	30.1	0.48
	7.5	283	0.1	0.65	0.31	3.6	0.35	60.6	0.31
	10.0	300	1.0	0.54	0.31	16.1	0.17	152.9	0.43
	10.0	300	0.1	0.45	0.24	5.0	0.27	78.2	0.47
water plus ions	7.5	300	0.1	0.74	0.43	4.1	0.29	34.6	0.26
exp^37^		283		~1.0		5.6			

*t*_sim_ is the simulation time for each rebinding trajectory, *τ*_*i*_ are the rebinding times and *a*_*i*_ are their respective weights. *a*_3_ is the static component.

**Figure 6:**
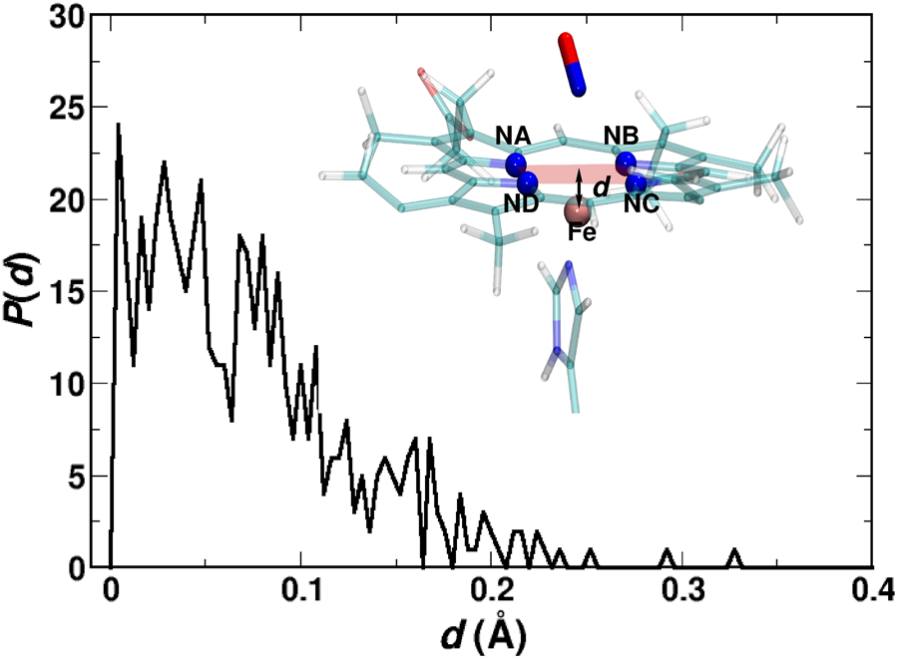
Distribution *P*(*d*) of the Fe-oop distance, *d*, at the moment of rebinding from the rebinding trajectories for MpNO in G/W mixture. The distance *d* is shown with double-headed black arrow in the inset. NA, NB, NC, ND and Fe atoms are shown in VDW representation.

In order to better understand how NO-rebinding occurs at a molecular level, the Fe-out of plane (Fe-oop) distance, *d* (i.e. the distance between the iron and the heme plane), is calculated at the moment of rebinding for each of the trajectories. Here, the heme plane is defined by the average plane through the four nitrogen atoms (NA, NB, NC and ND), see inset of Fig. 6. The distribution of these distances, *P*(*d*), is reported in Fig. 6. For 30% of the cases the heme-iron is further below the plane than 0.1 Å, i.e. in a domed structure. This is consistent with recent experimental work using sub-picosecond time-resolved resonance Raman and femtosecond transient absorption spectroscopy which finds that NO-rebinding in a number of globins (wild type and mutant Mb, Hb, dehaloperoxidase, Cytc) can occur to a “domed structure^20^. On the other hand, it has been proposed in earlier work on Mb using electronic spectroscopy and kinetic measurement that NO-rebinding is governed by “harpooning”, i.e. the unpaired electron of the NO-molecule “harpoons” the heme-iron back into the heme-plane for rebinding^19^.

Figure 5 and Table 1 indicate that NO rebinding to Mp-9 in the 90% G/W mixture occurs on the ps time scale which corresponds to geminate rebinding. This is likely because the initial Fe-N distances for all 500 rebinding trajectories are constrained to within 2.5–5 Å of the heme-iron due to the caged structure formed by the glycerol-water and glycerol-glycerol hydrogen bonding network (see Fig. 4) which makes geminate rebinding highly efficient. To corroborate this interpretation the translational motion of the NO ligand after photo-dissociation was considered. Figure 4d (blue) suggests that the typical distance between the heme-iron and the center of mass of the photodissociated ligand is ≈3.25 Å in the G/W mixture. The average speed of the NO-center of mass is between 2 and 12 Å/ps which indicates that the NO-ligand travels the 1.5 Å between the position after photodissociation and the bound state in 0.1 to 0.7 ps which is consistent with a rebinding time scale *τ*_1_ ranging from 0.55 to 0.64 ps. Hence, the sub-ps time scale is attributed to the geminate phase in which the ligand rebinds directly after photodissociation. The confined translational motion of the NO for few typical rebinding trajectories are shown in Section S1 of the Supporting Information (SI).

The effect of changing the asymptotic separation Δ is negligible for NO rebinding to Mp in the G/W mixture. While for Δ = 10 kcal/mol all the 500 trajectories rebind within 20 ps, 493 out of 500 trajectories geminately rebind into the MpNO conformation within 20 ps for Δ = 7.5 kcal/mol. In MbNO the recommended value to correctly capture rebinding time scales and rebinding fractions is 6.1 kcal/mol^21^. The remaining 7 trajectories form the isomer MpON (see below) which is stable for at least 1 ns for 5 trajectories. The other 2 trajectories isomerize back to MpNO within 1 ns. This suggests that the stability and occurrence of an MpON state depends on the value of Δ which is consistent with previous findings for MbNO for which Δ = 6.1 kcal/mol correctly captures rebinding times and fractions^21^.

### Photodissociation and Rebinding Kinetics of MpNO in pure water

The photodissociation of NO from Mp in pure water was followed using the same protocol as that for MpNO in the G/W mixture. After photodissociation the final Fe-N distances for 500 trajectories of deligated NO in pure water are shown in Fig. 4d (red). The initial Fe-N distances for the rebinding trajectories are more broadly distributed than for simulations in the G/W mixture and range from 2.5–8 Å. Also, in pure water the photodissociated ligand diffuses much further away from the heme-iron (see red trace in Fig. 4c), returns to the region around the active site (the heme-iron) before it finally diffuses away into the solvent after 400 ps.

Given this very different behaviour of the NO-ligand after photo-dissociation it is expected that the rebinding kinetics also differs appreciably for simulations in the G/W mixture and the pure water environment. The survival fraction of the rebinding trajectories are fitted to a multi-exponential decay ${\sum }_{i=1}^{2}\,{a}_{i}\,\exp (-t/{\tau }_{i})+{a}_{3}$ with a static component (*a*_3_). The fitting parameters are given in Table 1 and the raw data and the fits are graphically displayed in Fig. 5b for different values of Δ. Here, the static component, *a*_3_, represents the fraction of trajectories which rebind on a time scale longer than 1 ns. For Δ = 7.5 kcal/mol, the calculated rebinding fraction is 0.63 (63%) for 500 trajectories run up to 1 ns which compares with 52% for simulations run for 0.1 ns. Two time scales and a static offset were necessary to satisfactorily represent the survival fractions. For 500 simulations run out to 1 ns each the rebinding time scales were 10 or 15 ps (for geminate rebinding) and 200 or 152 ps (for rebinding from the solvent) for Δ = 7.5 or 10 kcal/mol, respectively. This reduces to 3 or 5 ps (for geminate rebinding) and 30 or 78 ps (for rebinding from the solvent) for Δ = 7.5 or 10 kcal/mol, respectively, from 500 rebinding trajectories each run for 0.1 ns (see Table 1). Experimentally, the rebinding of MpNO in water was found to be single exponential with a time constant of 5.6 ps^37^.

The computed rebinding fraction for NO rebinding to Mp in pure water differs appreciably from the experiment. One difference between computations and experiment is the composition of the solvent. While in the simulations pure water is used, the experiments include different ions (potassium phosphate, sodium dithionate and sodium nitrite) which may affect the solvent, ligand, and peptide dynamics. Also, while the experiments were carried out at 283 K the temperature in the simulations was 300 K.

To better understand these findings, additional simulations were carried out. First, the effect of temperature was considered by running simulations at 283 K from which we found an increase in the rebinding fraction by 13%. Equilibrium simulations of MpNO in pure water at 283 K and 300 K find that the structural RMSD of the peptide relative to the initial, energy minimized structure changes from 1.6 Å at low temperature to 2.8 Å at 300 K which supports the notion that the structural dynamics is damped at lower temperature. Furthermore, the water self-diffusion coefficient *D* increases by 75% (from 0.30 Å^2^ to 0.55 Å^2^) between 283 K and 300 K. This is consistent with experiments^43,44^ which find an increase of 50% for *D*. The increase of the rebinding fraction with lowering temperature was also previously discussed for CO rebinding to H_2_O-FePPIX (water-coordinated iron protoporphyrin IX) and where it was attributed to the structural change of the porphyrin ring due to heme-doming thermal motion which is different at the two temperatures^17^. The lowering of the rebinding fraction with increasing temperature indicates relaxation-induced increase of rebinding barrier via Fe-oop motion^17^ which is explicitly taken into account in the RKHS potential^34^. However, the rebinding fraction of 65% (i.e. 52% + 13%) still does not yield a near unit rebinding efficiency.

Next, the effect of adding ions to the solvent was assessed. The ions which are present in the experiment are expected to influence the H-bonding of the water solvent molecules^45^. Rebinding simulations with Δ = 7.5 kcal/mol at 300 K were performed for 0.1 ns each (500 trajectories) in the presence of 0.15 M NaCl ions (7 sodium and 6 chloride ions). The rebinding fraction was 0.74 which is an increase by 22% compared to the simulations without ions at 300 K (see Fig. 5 and Table 1). The overall effect of ions (+22%) and the lower temperature (+13%) in the experiments leads to a predicted rebinding fraction of close to 90% from 0.1 ns long simulations at 283 K. This prediction was explicitly tested by running 500 independent simulations at 283 K, each 0.1 ns in length with ions present which yield a rebinding fraction of 83% in very good agreement with 87% inferred from assuming additivity for the salt and temperature effect. These results are now also in quite good agreement with experiment^37^. Finally, accounting for the fact that longer (1 ns) simulations lead to an additional increase of the rebinding fraction by about 10% gives almost quantitative agreement with experiment when ions and the influence of lower temperature are included.

For further interpret the effects of Na^+^ and Cl^−^ ions in water on the rebinding kinetics compared to simulations in pure water, additional simulations and analyses were carried out. First, the structural dynamics of Mp-9 in the two environments was considered by computing RMSD relative to the initial energy minimized structure from 1.5 ns equilibrium simulations at 283 K and 300 K. Including the ions (RMSD = 2.1 Å) dampens peptide structural fluctuations compared to simulations in pure water (RMSD = 2.8 Å); likewise these fluctuations increase with increasing temperature in pure water from RMSD = 1.6 Å at 283 K to RMSD = 2.8 Å at 300 K, as expected. Furthermore, the distribution of ligand positions after photodissociation at 283 K was found to be damped for simulations in water with ions (average Fe-N distance is 3.60 Å) compared to the situation in pure water (3.85 Å). Hence, it is concluded that addition of ions dampens the structural dynamics of the peptide and decreases diffusional motion of the photodissociated ligand which leads to the increased NO-rebinding as observed in the explicit reactive MD simulations.

In order to correlate time scales and structural properties the rebinding trajectories for dissociated NO in pure water at 300 K (for Δ = 7.5 kcal/mol) are separated into three different clusters according to their rebinding time, i.e. 0 < *τ* < 20 ps (“short”), 20 < *τ* < 1 ns (“intermediate”), and *τ* > 1 ns (“long”) where the cluster 0 < *τ* < 20 ps indicates the cluster of trajectories for which NO rebinds within 0 and 20 ps. These time intervals approximately reflect the rebinding timescales *τ*_1_ and *τ*_2_ in Table 1.

**Figure 7:**
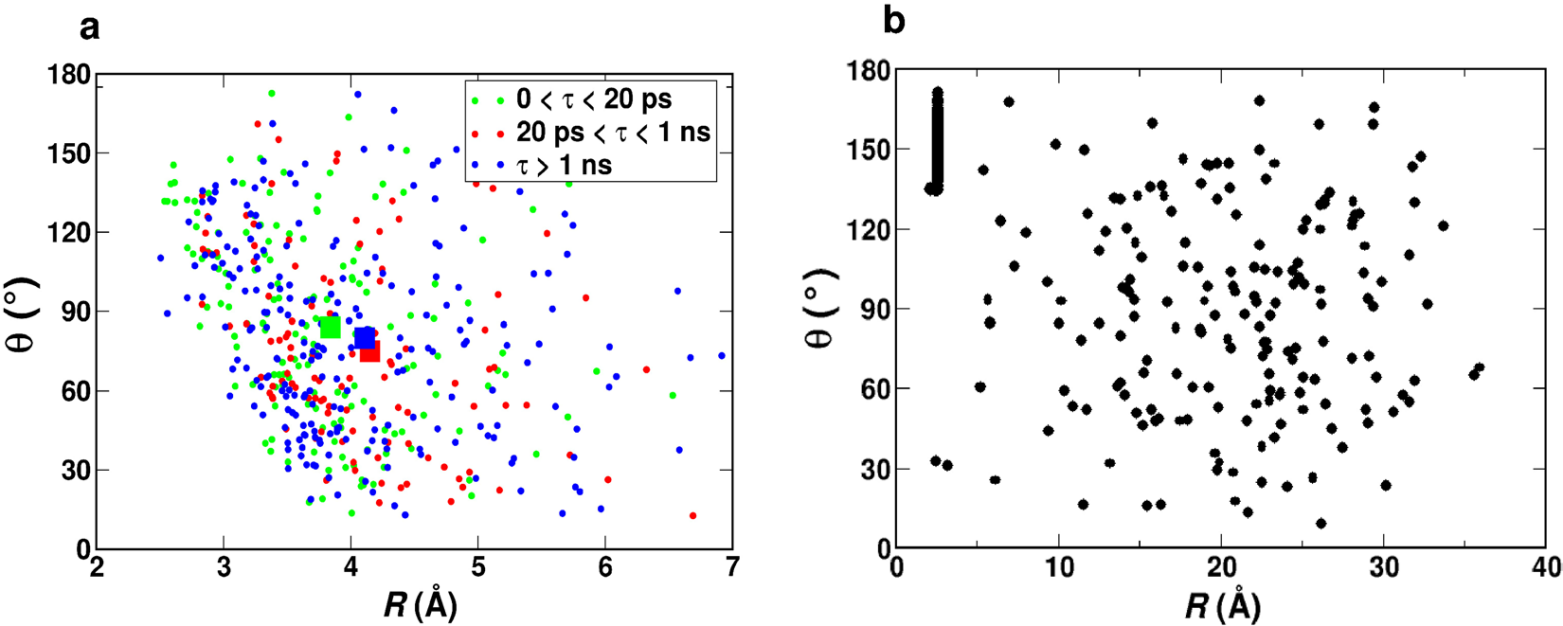
(**a**) The final *R* and *θ* for 500 trajectories after photodissociation clustered according to their rebinding time scales, *τ*, for MpNO in water for Δ = 7.5 kcal/mol. The big square of corresponding color refer to the centroid of each cluster; (**b**) Scatter plot of the final *R* and *θ* after 1 ns for 500 trajectories. The cluster of points (looking like vertical line) at *R* ~ 2.5 Å and *θ* ~ 130 − 170° correspond to the bound state.

Figure 7a shows the scatter plot of final *R* and *θ* values after photo-dissociation, right before rebinding starts, for each of the trajectories. Each point corresponds to the initial position of the rebinding trajectory in the 2-dimensional space of *R* and *θ*. 60% of the green points i.e. 0 < *τ* < 20 ps cluster, are situated close to the crossing (between ^4^*A* and ^2^*A* states) point (*R* < 4 Å) favouring the rebinding to happen within the time scale of few ps (“geminate rebinding”); see also the discussion above concerning geminate rebinding. All points from the other two clusters are distributed evenly in this two-dimensional space with ~50% of the points at *R* < 4 Å and in these cases, the rebinding time does not depend on the initial position of the NO. While 36% of the 500 trajectories rebind within 20 ps, 25% of them rebind within 1 ns. For the rest of the trajectories NO remained unbound within 1 ns.

It is also evident from the position of the centroid ([3.84 Å, 84°] for the green cluster, [4.15 Å, 75°] for the red cluster and [4.11 Å, 80°] for the blue cluster, filled square) of each cluster that the rebinding time scale depends on the initial position of the NO only for the shortest time scale i.e. the 0 < *τ* < 20 ps cluster. For the other clusters, since the trajectories have sufficient time to sample configurational space, the rebinding time scale cannot be distinguished based on the initial position of the NO and therefore the centroid for the clusters with longer time scales (red and blue squares) was found to be close to each other. To compare with the results in G/W mixture, an analysis for rebinding within 1 ps was also done. Only 7.6% (85% of them being close to the crossing point) of the total trajectories was found to rebind within 1 ps for MpNO in pure water compared to 80% for MpNO in the G/W mixture (see Table 1).

The scatter plot of final *R* and *θ* for all 500 rebinding trajectories is shown in Fig. 7b. The cluster around *R* ~ 2.5 Å and 130° < *θ* < 160° corresponds to the reactive trajectories that rebind (63%), the remaining (scattered) 37% of the events correspond to non-reactive trajectories. The movement of NO in the *R* − *θ* space for few typical rebinding trajectories in pure water are shown in the SI (Section S2).

### MpON to MpNO isomerisation kinetics in different environments

For several model compounds it has been found that NO can bind to the heme group in two conformations: FeNO and FeON^46^. As earlier^47^ computations proposed the possibility for these two binding modes to exist but more recent computations^21,34^ and experiments were unable to substantiate them^48^. It is of interest to consider the existence of a metastable MpON state for Mp-9. The fact that experimentally, MbON was not found was traced back to the energetic ordering of the ^2^*A* and ^4^*A* states which favours an NO-unbound state for configurations corresponding to the MbON state. Hence, we set out to investigate the possibility to stabilize the FeON state in Mp-9. For this the MpON → MpNO isomerisation kinetics was followed in the gas phase, in the G/W mixture and in pure water.

From a 3 ns *NVE* simulation with the system in the FeON structure (harmonic FeON angular potential and constrained Fe-O distance), 500 snapshots were extracted every 5 ps. Then, individual trajectories were run for 1 ns on the ^2^*A* surface which allows isomerization. The survival fraction of the MpON state was determined and follows a single-exponential decay *a*_1_ exp(−*t*/*τ*_1_). Isomerisation fraction and isomerisation time scales for the MpON to MpNO isomerisation in the gas phase and in pure water are tabulated in Table 2.

**Table 2:** Parameters for the single-exponential fit of the survival fraction for MpON to MpNO isomerisation trajectories in gas phase and in pure water.

Environment	isomerized fraction	a_1_	*τ*_1_ (ps)
gas phase	0.78	0.75	250.1
In water	0.70	0.94	836.0

Both, in the gas phase and in water, only one time scale was found for the isomerisation. The process is slower in water (836 ps) than in the gas phase (250 ps). The isomerization fraction on a time scale of 1 ns is 70% and 78% in water and the gas phase, respectively, see Table 2. For comparison, 200 trajectories were also run in the G/W mixture and the isomerisation fraction was found to be negligible (0.025). This indicates that the isomerisation barrier is higher in the G/W mixture (most probably due to the packing of Glycerol around MpON leading to the higher Mp-ON to Mp$\cdots$NO barrier) than in water and in the gas phase which is consistent with the potential energy cut shown in Fig. 3.

## Conclusion

In this work, the rebinding kinetics of MpNO in G/W mixture, pure water and water containing ions was studied using molecular dynamics simulations with reactive force fields based on a RKHS representation of the *ab initio* data. The ligand dynamics as well as rebinding kinetics are found to be significantly different in G/W mixture, pure water and in water plus ion environment. While for the 90% G/W mixture rebinding is geminate, the rebinding fraction decreases to 63% in pure water. The disagreement with the experimentally^37^ determined rebinding fraction of close to 100% can be explained by the fact that in the experiments the ions present in the solvent lead to structuring of the solvent akin to a G/W mixture. A somewhat related effect was observed for dioxygen interacting with hemoglobin for which the heterogeneous distribution of the diatoms in the water environment influence the protein dynamics and may have an effect on O_2_ binding^49^. The rebinding time scales in G/W are sub-picosecond and ~5 ps whereas in pure water they are longer with a pronounced offset (unbound fraction). For G/W the computed rebinding time scale differs from experiment (8 or 11 ps) by about a factor of 1.5–2 which is consistent with what is known from NO rebinding to Mb^12,21,50–52^.

In summary, it is found that NO rebinding to Mp-9 is affected by the surrounding solvent structuring. Using state-of-the art interaction potentials, the behaviour in a G/W mixture can be faithfully captured whereas in pure water the differences with experiment provides important insight into the role of ions in structuring the solvent and providing a stabilizing environment that promotes rebinding. This is of particular importance for solvent-exposed active sites. The effect of solvent structuring and crowding relevant for the molecular dynamics in cells can therefore be expected to be amenable to atomistic simulations and should be explored more rigorously when moving from single-protein to more realistic multi-protein, cellular-like environments.

## Methods

The initial structure for the present simulations is that of microperoxidase 9 (Mp-9) for which an X-ray structure is available (PDB code 3M4C)^53^. For Mp-8 no X-ray structure is available which is the motivation to work with Mp-9. Both sequences agree in that they contain a Cys-X-X-Cys-His motif in which the heme-iron is bound to the histidine side chain and the two cysteine-residues are covalently bound to the heme-framework, see Fig. 1. The sixth available site of the heme-iron is occupied by the NO-ligand which is solvent-exposed. However, the overall sequence of Mp-8 (CAQCHTVE) differs from that of Mp-9 (KTTCNACHQ). Three states are relevant in the present work which are referred to as MpNO (FeNO), MpON (FeON) and Mp$\cdots$NO (photodissociated NO).

In Mp-9 (see Fig. 1) the peptide chain with the three covalent bonds (one with His8 and two with two cysteine residues) holds the heme group like an inverted basket handle. The heme-group and the NO ligand are completely exposed to the surrounding solvent for MpNO which makes this system very different from MbNO or Cyt-cNO for which the protein-active site is encapsulated by the protein framework and access of individual solvent molecules is difficult.

### Intermolecular Interactions

The total potential energy, ${V}_{{\rm{tot}}}(\vec{X})$ of the system is written as

1 $${V}_{tot}(\vec{X})={V}_{{\rm{FF}}}(\vec{Q})+V(R,\theta ,\varphi )$$

where, ${V}_{{\rm{FF}}}(\vec{Q})$ is the energy for all degrees of freedom except for the coordinates (*R*, *θ*) which describe the position of the NO-ligand relative to the heme-Fe, and the Fe-out-of plane position *ϕ*. For ${V}_{{\rm{FF}}}(\vec{Q})$ the standard CHARMM22 force field^54^ is used whereas *V*(*R*, *θ*, *ϕ*) is a reproducing kernel Hilbert space (RKHS) representation of energies calculated at the B3LYP/6-31G(d,p) level of theory^34^. The coordinates (*R*, *θ*, *ϕ*) (see Fig. 1) are the Fe-CoM_NO_ distance *R*, the Fe-COM_NO_-O angle *θ* and the Fe-doming coordinate *ϕ* which describes the energy associated with the Fe moving relative to the average porphyrin plane defined by the 4 nitrogen atoms bound to the metal.

For studying the ligand-bound and photodissociated states a suitable description of the intermolecular interactions for the ^2^*A* and ^4^*A* states is required. For this, the previously devised reactive potential energy surfaces based on a reproducing kernel Hilbert space (RKHS) representation were used^21,34^. The basis for these PESs are energies for several thousand configurations determined at the B3LYP/6-31G(d,p) level of theory on a 3-dimensional grid. The energies *V*(*R*, *θ*, *ϕ*) were represented as

2 $$V(R,\theta ,\varphi )=\sum _{\lambda =0}^{10}\,{V}_{\lambda }(R,\varphi ){P}_{\lambda }(\cos \,\theta )+{V}_{c}(\varphi )$$

where *P*_*λ*_ (cos *θ*) are Legendre polynomials and ${V}_{c}(\varphi )=\tfrac{1}{2}k{(\varphi -{\varphi }_{e})}^{2}$. The radial strength functions *V*_*λ*_(*R*, *ϕ*) are represented as a reproducing kernel^33^

3 $${V}_{\lambda }(R,\varphi )=\sum _{i,j}\,{\beta }_{\lambda ,i,j}\cdot \kappa (R,{R}_{i})\cdot {\rm{\Gamma }}(\varphi ,{\varphi }_{j})$$

where *κ*(*R*, *R*_*i*_) is a radial reproducing kernel and Γ(*ϕ*, *ϕ*_*j*_) is a Gaussian reproducing kernel. Until this point the energies of the two states obtained are independent. However, as soon as the two PESs are mixed in MS-ARMD simulations their zeroes of energy must be related to one another such that the two energy levels maintain their correct ordering in the asymptotic region (*R* → ∞). This is achieved by adding a scalar constant Δ to the ^4^A state in the present case^21,40,55^.

### Molecular Dynamics Simulations

Molecular Dynamics (MD) simulations were carried out for Mp-9 in G/W mixture, pure water and water plus ion environments. The pure water and water plus ions solvent box was a cubic box of size 41^3^ Å^3^ with pure water and water plus 7 sodium and 6 chloride ions (0.15 M NaCl), respectively and the other one a mixed (90/10 per volume) glycerol/water (G/W) mixture. All MD simulations (unless stated otherwise) were carried out with CHARMM^56^ using periodic boundary conditions, a time step of Δ*t* = 1 fs, a nonbonded cut-off of 12 Å and in an *NVE* ensemble. Starting from the solvated X-ray structure, the solvent was minimized, heated and equilibrated keeping the protein-ligand system fixed to release bad contacts. Then the full system was minimized, heated (300 K) and equilibrated (for 200 ps) in the *NVT* ensemble and further equilibration was carried out in the *NpT* ensemble.

Photodissociation was induced by invoking the “sudden approximation”^13^ in which for a given ground state configuration (^2^*A*) the Fe-NO bond is broken and the system is propagated on the excited state (^4^*A*). For this, a 3 ns long *NVE* simulation was performed in the ^2^*A* state and coordinates and velocities were saved every 5 ps to obtain a total of 500 initial conditions from the last 2.5 ns of simulation. Since in a physical experiment the photodeligation process is ultrafast (300–500 fs)^14^, each of the 500 trajectories were propagated for 500 fs on the ^4^*A* deligated state and the final coordinates and velocities were used as the initial condition of the rebinding simulations. The rebinding simulations were run using MS-ARMD^21,34,35^ which mixes potential energy surfaces depending on their relative energy and ensures smooth dynamics around crossing region.

## Electronic supplementary material

Supplementary Information
