# Supplementary material for: Solvent Composition Drives the Rebinding Kinetics of Nitric Oxide to Microperoxidase
Source: Sci Rep. 2018 Mar 27;8:5281. doi: 10.1038/s41598-018-22944-z (PMC5869715; doi:10.1038/s41598-018-22944-z)
Supplement: Supplementary file 1 — Supplementary Information [file 41598_2018_22944_MOESM1_ESM.pdf]

Supporting Information
Solvent Composition Drives the Rebinding Kinetics of Nitric Oxide to Microperoxidase

Padmabati Mondal and Markus Meuwly

Department of Chemistry, University of Basel, Klingelbergstrasse 80, 4056- Basel, Switzerland

February 23, 2018

S1: Movement of NO in the $R-\theta$ space for MpNO rebinding in G/W mixture

To show the hindered translational motion of NO within the cavity created by caged structure due to G/W and G/G hydrogen bonding, the NO coordinates for few typical rebinding trajectories with different rebinding time are projected in the $R-\theta$ space.

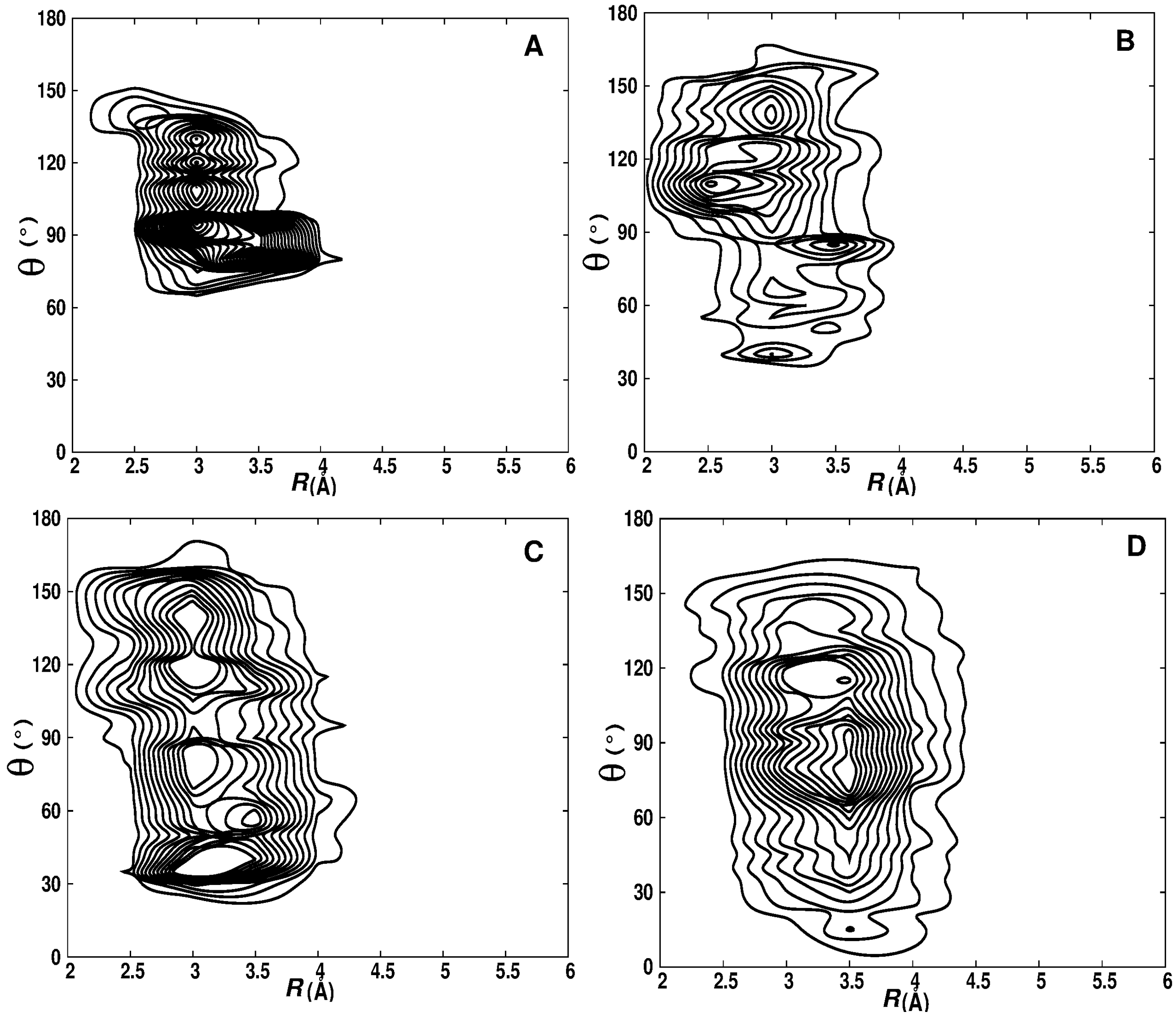

Figure S1: Contour plots for the probabilities of few typical rebinding trajectories for MpNO in G/W mixture for $\Delta = 10$ kcal/mol, with (A) $\tau = 0.6$ ps,(B) $\tau = 5$ ps, (C) $\tau = 20$ ps and (D) $\tau = 50$ ps, projected in the $R - \theta$ space.

Figure shows the contour plots for probabilities of a few typical trajectories for the rebinding of MpNO in G/W mixture projected on the $R - \theta$ space. In all cases, the trajectories are confined within $2.5 < R < 5.0$ which indicates that unlike the case of MpNO in water, NO in G/W mixture cannot diffuse into the solvent due to the caged structure formed by the hydrogen-bonding between water and glycerol. The final R and θ for all the total 500 trajectories for $\Delta = 10$ kcal/mol were found in the range of $2.4 - 2.55$ Å and $130° - 160°$, respectively.

S2: Movement of NO in the $R - \theta$ space for MpNO rebinding in pure water

For comparison, the movement of NO for the rebinding trajectories of MpNO in pure water are also shown (as for the G/W mixture) in the $R - \theta$ space.

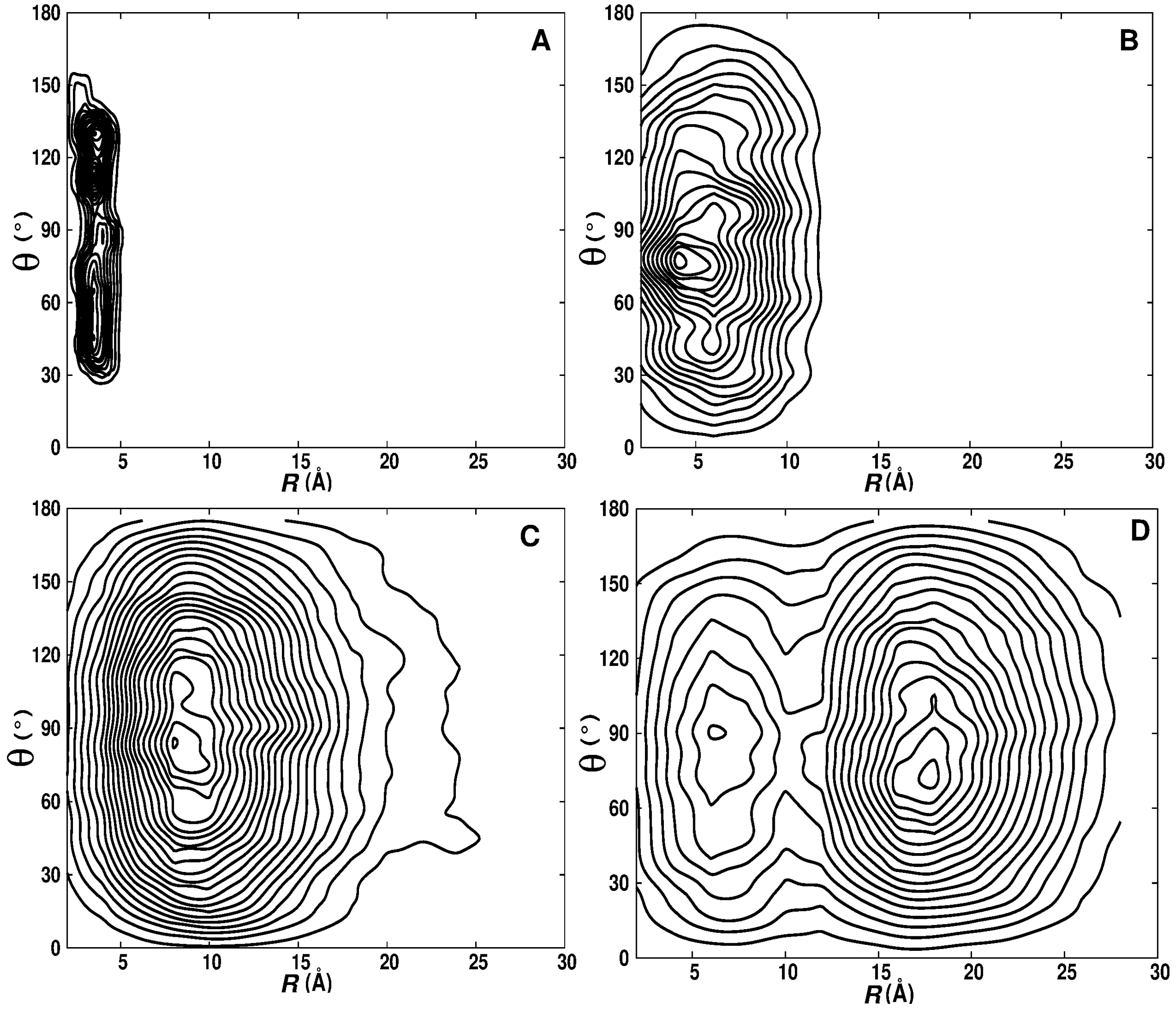

Figure S2: Contour plots for the probability of few typical rebinding trajectories for MpNO in pure water for $\Delta = 7.5$ kcal/mol, with (A) $\tau = 5$ ps ,(B) $\tau = 52$ ps, (C) $\tau = 537$ ps, and (D) $\tau = 944$ ps, projected in the $R - \theta$ space.

Figure shows contour plots for the probabilities of few typical rebinding trajectories for MpNO in pure water projected in the $R - \theta$ space with corresponding rebinding time, τ. It was found that the trajectories corresponding to $\tau \geq 20$ ps moves far along the R coordinate

following the diffusion of NO in water.
